# The application of a novel hydrodynamic cavitation device to debride intra-articular monosodium urate crystals

**DOI:** 10.1186/s12893-023-01929-4

**Published:** 2023-02-10

**Authors:** Hanlin Xu, Shengkun Li, Ling Cao, Xiaoxia Zhu, Yu Xue, Yu Huang, Yinghui Hua

**Affiliations:** 1 Department of Sports Medicine, Huashan Hospital, Fudan University, Shanghai, 200040 China; 2 Department of Rheumatology, Huashan Hospital, Fudan University, Shanghai, 200040 China; 3 State Key Laboratory of Mechanical System and Vibration, School of Mechanical Engineering, Institute of Vibration Shock and Noise, Shanghai Jiao Tong University, 800 Dongchuan Road, Minhang District, Shanghai, 200240 China

**Keywords:** Gout, Monosodium urate, Cavitation device, Intra-articular deposition

## Abstract

**Introduction:**

Efficient and complete debridement of intra-articular deposits of monosodium urate crystals is rarely achieved by existing arthroscopic tools such as shavers or radiofrequency ablation, while cavitation technology represents a prospective solution for the non-invasive clearance of adhesions at intra-articular interfaces.

**Methods:**

Simulation modeling was conducted to identify the optimal parameters for the device, including nozzle diameters and jet pressures. Gouty arthritis model was established in twelve rats that were equally and randomly allocated into a cavitation debridement group or a curette debridement group. A direct injection nozzle was designed and then applied on animal model to verify the effect of the cavitation jet device on the removal of crystal deposits. Image analysis was performed to evaluate the clearance efficiency of the cavitation device and the pathological features of surrounding tissue were collected in all groups.

**Results:**

To maximize cavitation with the practical requirements of the operation, an experimental rig was applied, including a 1 mm direct injection nozzle with a jet pressure of 2.0 MPa at a distance of 20 mm and a nitrogen bottle as high-pressure gas source. With regards to feasibility of the device, the clearance rates in the cavitation group were over 97% and were significantly different from the control group. Pathological examination showed that the deposition of monosodium urate crystals was removed completely while preserving the normal structure of the collagen fibers.

**Conclusions:**

We developed a promising surgical device to efficiently remove intra-articular deposits of monosodium urate crystals. The feasibility and safety profile of the device were also verified in a rat model. Our findings provide a non-invasive method for the intraoperative treatment of refractory gouty arthritis.

## Introduction

Gout is a common metabolic disease characterized by joint pain, swelling and mechanical dysfunction [1]. Deposition of monosodium urates (MSU) crystals on the surface of cartilage, meniscus, synovium, ligament and tendons triggers gout flares under certain conditions (such as low temperature, fatigue, trauma), and then progress to irreversible structural damage [2, 3, 4].

Although the administration of medications for controling the serum levels of uric acid and relieving specific symptoms is recommended as a first-line strategy for patients with chronic gouty arthritis, surgery is still an effective method for those with a large number of crystal deposits or those with joints exhibiting severe structural or functional effects [5, 6]. Over recent years, arthroscopic debridement has become a common surgical method for the removal of crystals in multiple joints; this is due to the fact that arthroscopy is a less invasive approach, provides a greater visual field, and is associated with a reduced risk of complications and a faster duration of rehabilitation [7]. However, extensive and tight adhesion between intra-articular structures, and the deposition of crystals, makes regular arthroscopic tools such as the spatula or curette very difficult and time-consuming to achieve a satisfactory clearance outcome. In addition, iatrogenic injuries to the adjacent joint structures may lead to pain, delayed recovery, or other complications [8, 9]. Therefore, there is a clear desire to develop a new method for the rapid debridement of MSU crystals at joint interfaces without damaging adjacent tissues to optimize surgical treatment for gouty arthritis.

Cavitation is defined as the generation, growth and collapse of cavities with sufficient liquid pressure drop and has been developed over recent decades for application in the ship industry, aviation, metallurgy, machinery and medical field [10]. The mechanical effect of it is ascribed to stable cavitation caused by continuous microbubble oscillation and inertial cavitation generated by microbubble bursting [11]. Currently, cavitation technology was comprehensively applied to diagnosis and surgical ablation in clinical practice [10, 12]. Hydrodynamic cavitation is defined as cavitation generated in flow. Utilizing the energy generated by the cavitation process and the impact of the shock wave associated with the highly concentrated micro jet, the impact pressure on an object surfaces could be used for the purpose of cleaning [11, 13]. Hence, the cavitation water jet represents a mature form of industrial engineering technology that is a potential solution the clinical problems associated with the clearance of intra-articular MSU deposition.

In the present study, we determined the appropriate power and accuracy of this technology and then developed a miniaturized cavitation jet system that could be used to clean intra-articular deposits of MSU. Simulation modeling allows us to identify the ideal nozzle diameters and jet pressures. Then, we tested joint specimens from rat models of gout under saline immersion to mimic the normal working condition of the arthroscopy. Using the model, we compared the coverage fraction of crystal deposition on articular surfaces and performed histological evaluations before and after debridement. These analyses confirmed that the new hydrodynamic cavitation device was safe and effective for the clearance of MSU crystal precipitation and did not cause damage to structures of the joint. Our research provides an innovative and minimally invasive approach for the removal of MSU deposits with high surface clearance and safe outcomes for patients with refractory gouty arthritis.

## Materials and methods

### Animal study

A total of twelve male Sprague–Dawley (SD) rats (Shanghai Laboratory Animal Research Center, Shanghai, China) aged 8 weeks and weighing 160 to 180 g were used as a research model in the current research. Experimental procedures were approved by the Animal Welfare and Ethics Group, Department of Experimental Animal Science; Shanghai Medical College of Fudan University, Shanghai, China (Approval Number: 2019020405). The preparation of MSU crystals and the establishment of an animal model of gout are in accordance with a previous study [14].

Tissue samples were harvested from knee joints on the five days after model was established [14]. The knee joints of rats (n = 24) were divided into an cavitation debridement group (n = 12) and a curette debridement group (n = 12). For both groups, specimens were dipped in 0.9% saline for 4 h to remove MSU crystals that were distributed in the joint cavities instead of being deposited on the joint structures prior to the application of the cavitation device or arthroscopic tools.

### Simulation modeling

**Fig.1:**
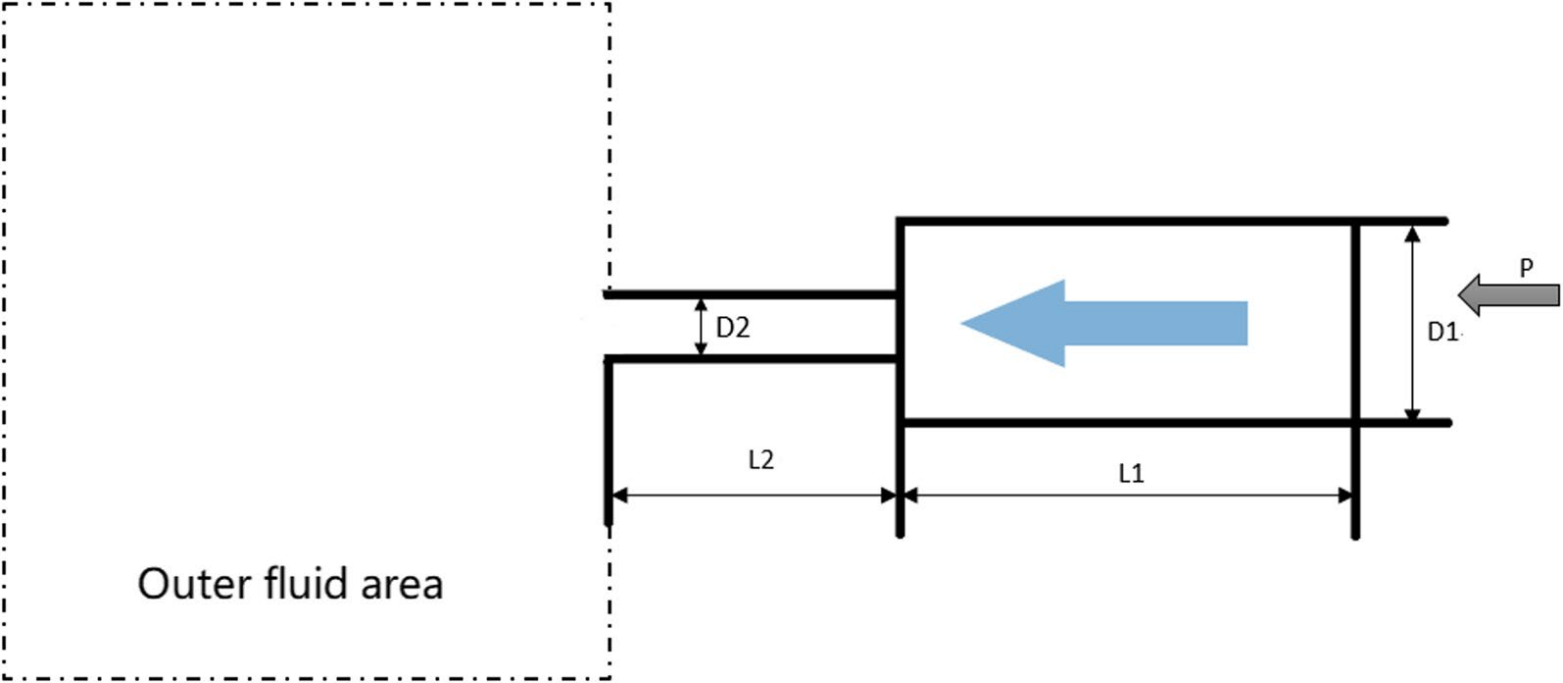
Design of geometry structure of the nozzle

Simulations were performed in the ‘Fluent’ module on Ansys Workbench® version 17.0(Ansys, PA, USA). Using this platform, we investigated the effects of different nozzle diameters and jet pressures on the cavitation effect to optimize parameters for treating gout crystallization, including the geometry of the nozzle, and the experimental pressure. We set a single distance of 2 cm between the nozzle and surface under comprehensive consideration, i.e., having a relatively small pressure compared to the position with a shorter distance from the nozzle and a relatively large velocity compared to that with a longer distance. Figure 1 shows the geometrical structure of the nozzle.

Using Fig. 1 as a guide, we set the large diameter D1 to 10 mm, the length of the large tube L1 to 100 mm, the length of the nozzle L2 to 50 mm, and the outer fluid area to a cylindrical fluid domain with a diameter of 0.4 m and a length of 0.8 m. Simulation parameters relating to the diameter of the nozzle and jet pressure are shown in Table 1.

**Table 1:** Simulating parameters of diameter of nozzle and jet pressure

Simulation case	Diameter of nozzle D2/mm	Jet pressure /MPa	Nozzle length/mm
1	1	1.0	50
2	1	1.5	50
3	1	2.0	50
4	2	1.0	50
5	2	1.5	50
6	2	2.0	50

**Fig.2:**
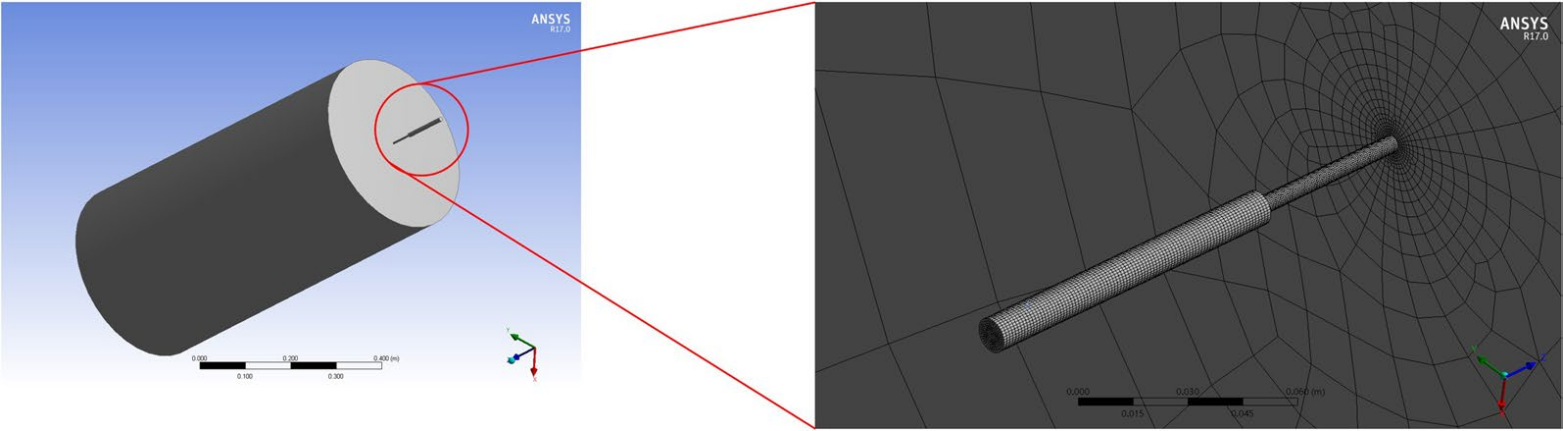
Diagram of fluid domain (left) and multi-zone grid (right)

Taking the diameter of the nozzle as an example, the fluid flow of the nozzle was complex and required grids of both high-density and high-quality. In contrast, the outer fluent area's mesh was divided into a relatively rough grid due to its relatively large size and simple fluid flow. Therefore, we adopted a multizone mesh division with a high-density grid for the area near the nozzle and a low-density grid for the outer flow area(Fig. 2). The parameters are as follows: 204,945 cells, 218,204 nodes, 0.30339 max orthogonal skew, and min orthogonal quality 0.69667.

To simulate the cavitation effects of the flow, Fluent module with the basic settings were used as follows: (1) Selecting the Eulerian model in the Multiphase flow with the salty water brine as the initial phase and the water vapor as the second phase. Setting the cavitation model to Schnerr and Sauer cavitation model. (2) Selecting the Realizable k-ε turbulence model. Wall Function Wall Function is set by default. (3) Setting the nozzle inlet as the pressure boundary condition with a value of the jet pressure, and the volume fraction of Phase 2 to 0. Set the nozzle outlet as the pressure boundary condition with a pressure of 0 Pa and the volume fraction of Phase 2 to 0. 4) Selecting the coupled solver solution method with Hybrid Initialization.

### Experimental apparatus

**Fig. 3:**
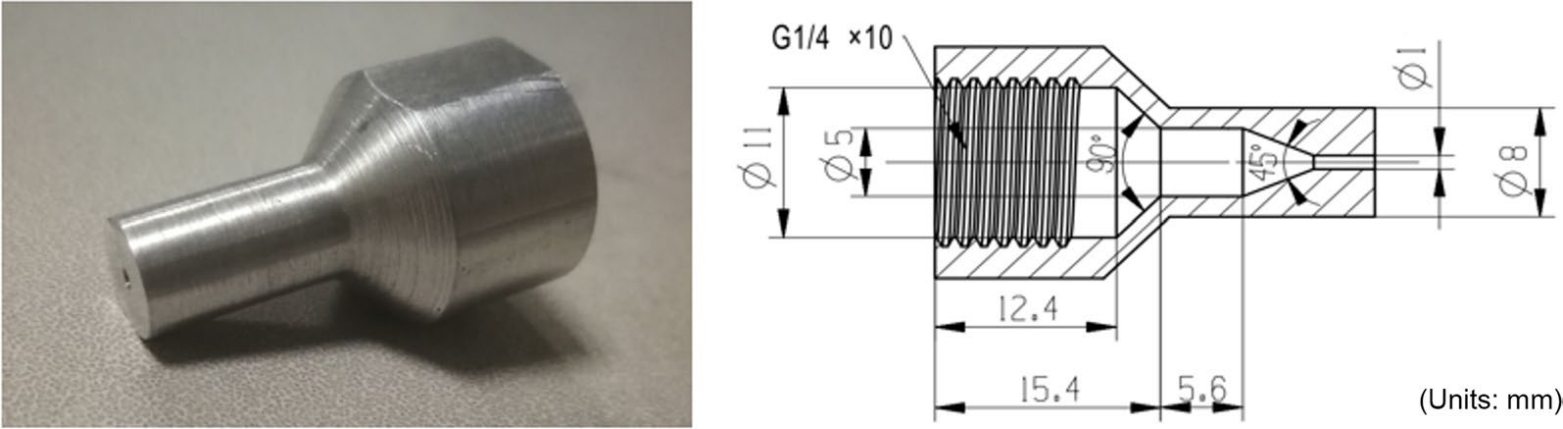
Diagram of nozzle structure

**Fig. 4:**
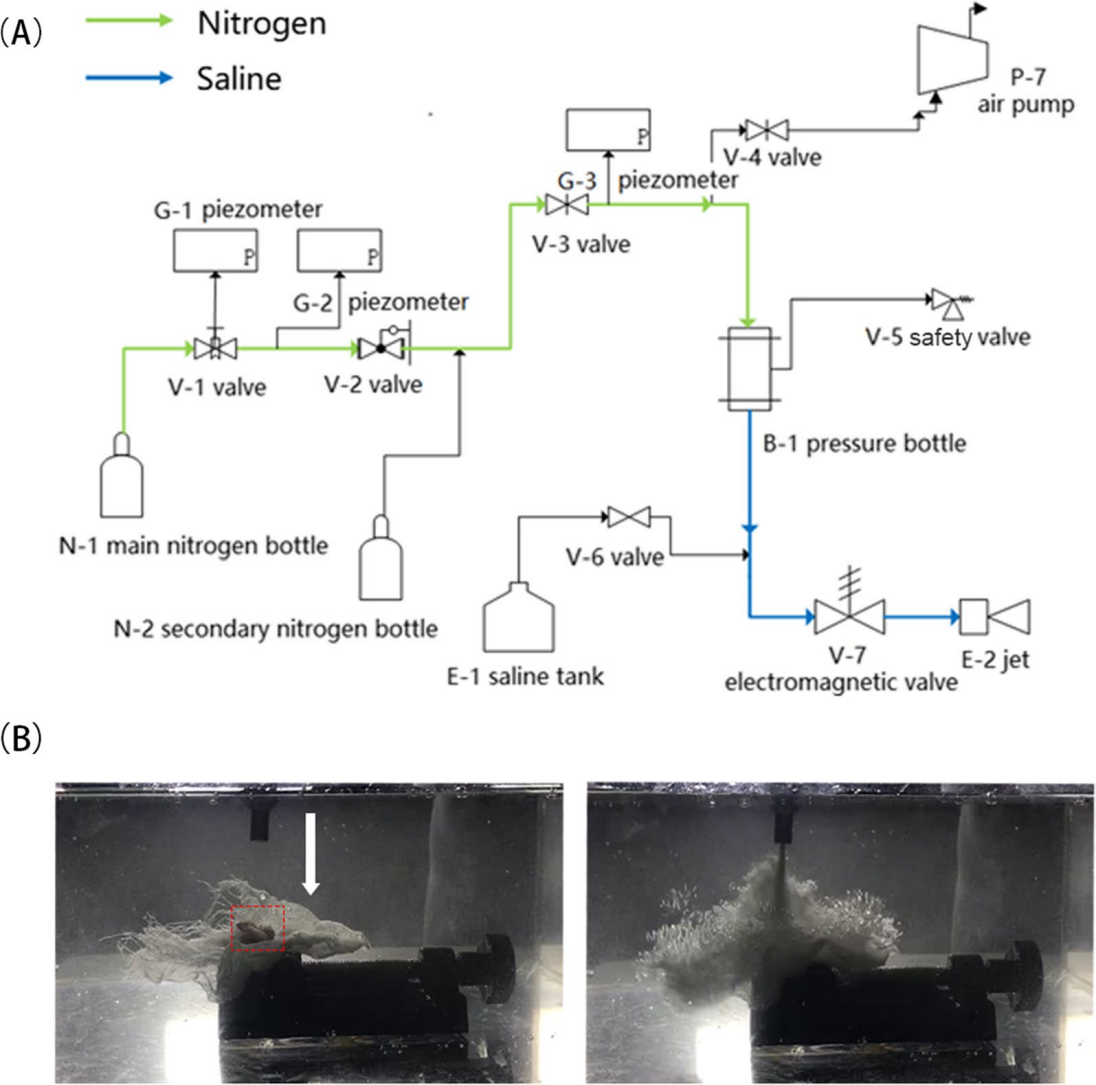
Schematics of the experimental rig design. Scheme in **A** costs less and is relative safe compared to plunger pump, and can be used as a laboratory feasibility test. In this scheme, nitrogen bottle is used as high pressure gas source, and other gas sources can be used instead. **B** Photos displays the joint fixed in experimental rig. Left, white arrow: direction of jet; Red dotted sqaure, the location of sample. Right, representative image of the cavitation tests on sample

The structure of the nozzle is shown in Fig. 3 and the experimental rig is shown in Fig. 4. To avoid potential damage to the intraarticular structures of the joint, the jet pressure at the nozzle was strictly controlled to below 2 MPa. A nitrogen bottle is used as high-pressure gas source for its advantages of low cost and safety compared to plunger pump.

### Debridement of intra-articular monosodium urates crystal

Simulations were performed to determine the optimal experimental parameters that meet the requirements of cavitation rate during the experiment. We then conducted experiments based on combination of simulation results and request of safety consideration to explore the effectiveness of the cavitation removal of crystal deposition.

A 1 mm direct injection nozzle, as shown in Fig. 3, was applied to investigate the best parameters for the cavitation jet device with regards to the removal of MSU crystal deposits. For experiments, the nozzle was submerged, the jet pressure varied from 0.7 to 2.0 MPa, the duration varied 5 to 30 s, and the distance between the nozzle jet and the surface of the sample was 20 mm.

Once the optimum parameters had been determined, we performed quantitative experiments. For the control group, the joint surfaces of rats were cleaned with a curette by senior surgeons. For the experimental group, cavitation jet tests were performed in both a qualitative and quantitative manner. The experimental parameters were as follows: the nozzle was submerged; the jet pressure was 2.0 MPa and the distance between the nozzle and the sample was 20 mm. Since deposition of crystals was unevenly on surface of joint, the duration for each deposition sites varied from 5 to 10 s for each target area, as recommended by an experienced surgeon, to control the total operative time. All pre- and post-intervention photos were acquired with a digital Single Lens Reflex camera (Canon, Japan) and analyzed using ImageJ software; the clearance ratio was defined as crystal pixels/joint area of pixels.

### Histologic examination

The synovium from rats were processed for H&E staining and Masson staining as previous described [15]. The required reagents were purchased from Sigma Chemical Co.St.Louis, MO, USA and Servicebio, Wuhan, China.

### Statistical analysis

Statistical analyses were performed by GraphPad Prism 8.0.1 software. All datasets were tested for normality for t-test, and if the normality test failed, the Mann Whitney rank-sum test was used for intra-group comparison. Results are expressed as mean ± SD. P value < 0.05 is considered as significant.

## Results

### Simulation modeling

**Fig. 5:**
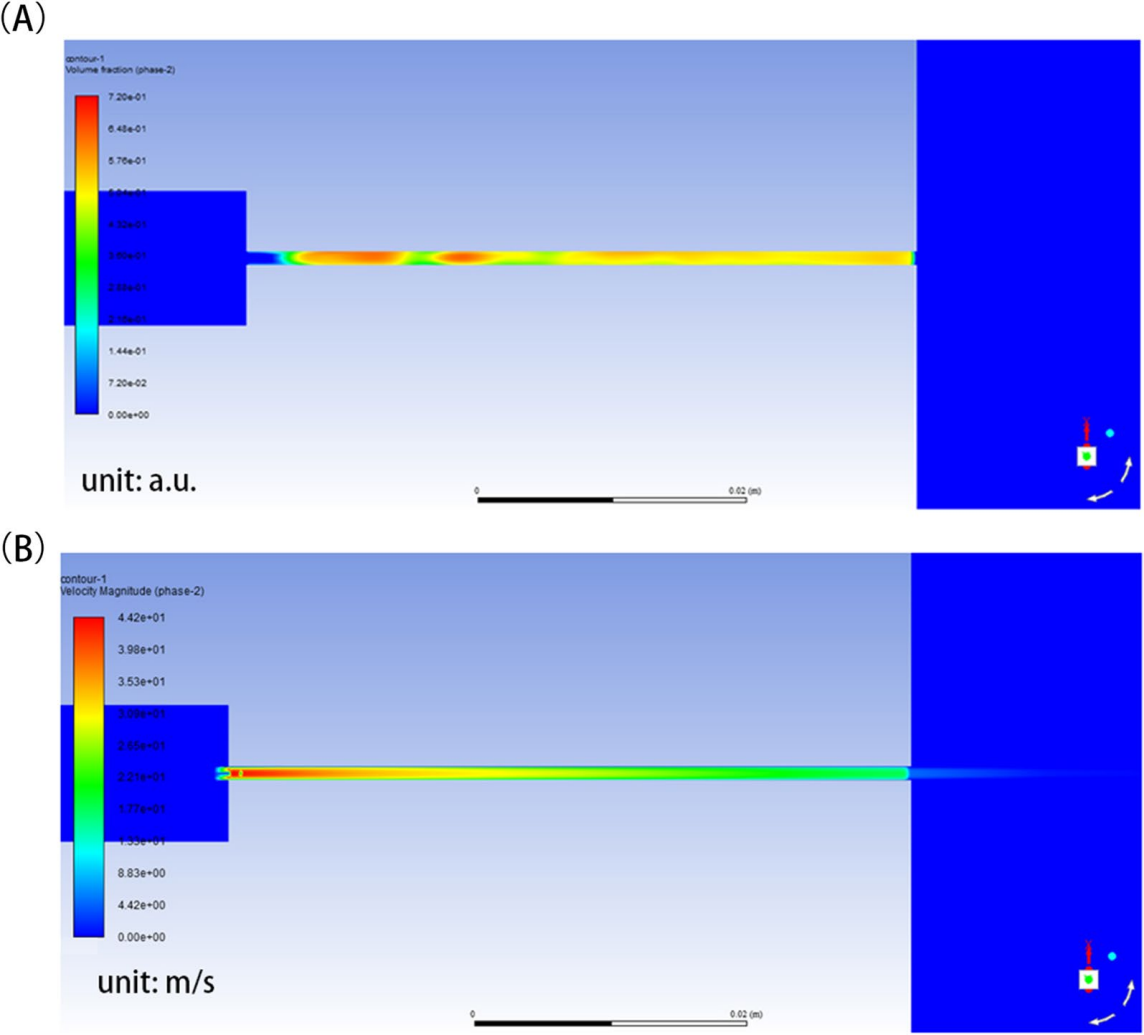
Diagram water vapor phase volume fraction and velocity cloud map.** A** Diagram vapor phase volume fraction nephogram (Diameter: 1 mm, Pressure: 1.0Mpa locally). **B** Diagram vapor phase velocity nephogram (Diameter: 1 mm, Pressure: 1.0Mpa locally)

**Fig. 6:**
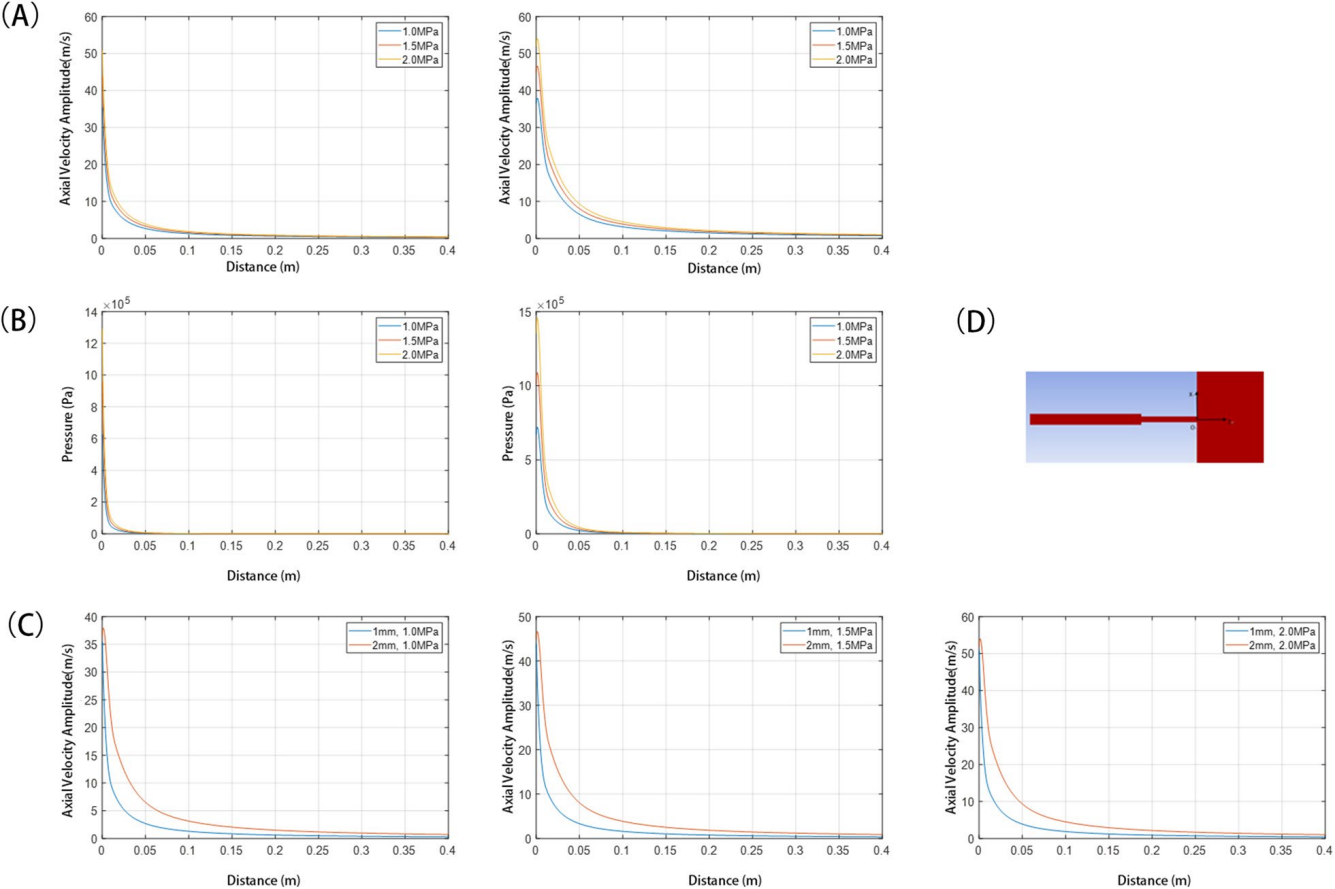
The velocity and pressure distributions of different sizes and pressures of nozzle. **A** Z axis velocity distribution curve under different jet pressure with nozzle diameter of 1 mm(left) and 2 mm(right). **B** Z axis pressure distribution curve under different jet pressure with nozzle diameter of 1 mm(left) and 2 mm(right). **C** Z axis velocity distribution curve under different nozzle diameters.(left:1.0Mpa, middle:1.5Mpa, right:2.0 MPa)** D** Coordinate system diagram

Figure 5 shows cloud maps of the water vapor phase volume and the velocity of the water vapor phase when using a nozzle with a 1 mm diameter and a jet pressure of 1.0 MPa. It was evident that the cavitation phenomenon occurred mainly inside the tube but decayed rapidly after entering the outer fluid area, both in terms of volume fraction and flow rate. The velocity and pressure distributions for different nozzle sizes and pressures are shown in Fig. 6.

Analysis showed that the flow velocity at the nozzle varies little with the nozzle diameter, but the decay velocity in the outer flow area would be slower with the larger nozzle diameter. The results also show that abrupt change in cross-section is conducive to cavitation, e.g. 1 mm nozzle has a larger vapour volume fraction than nozzles with a 2 mm diameter, and the cavity bubble spreads a wider range with the larger diameter of the nozzle.

### Efficacy and safety profile of the cavitation device

**Fig. 7:**
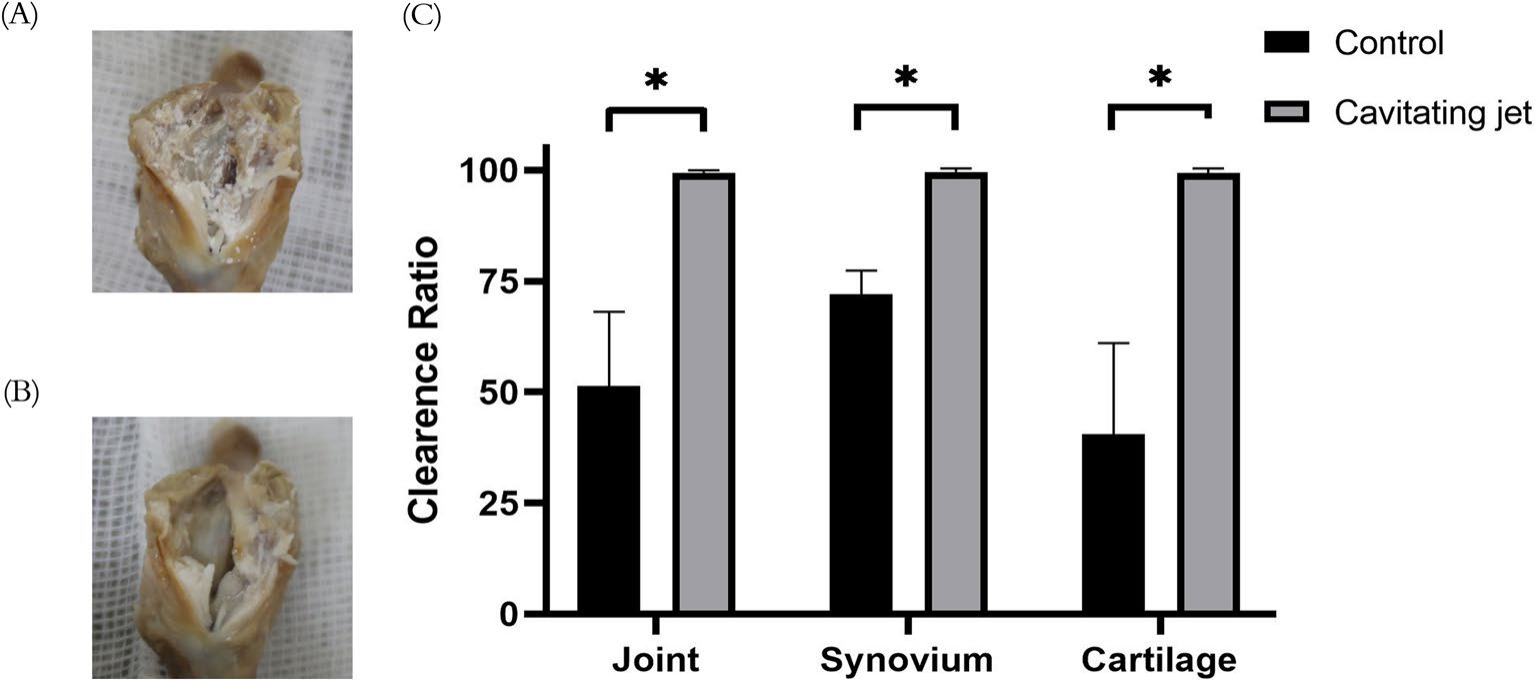
Clearance efficiency of cavitation device to joints from rat model. **A**, **B** Representative images of rat joints before and after hydrodynamic cavitation tests. Chalky tissues attached to surface of cartilage and synovium were removed by device. **C** Comparison of clearance efficiency between hydrodynamic cavitation group and control group. (*n* = 6 for each group; two-tailed t-test was applied; *P < 0.05. Data shown as mean ± s.e.m

Gross appearance and quantitative image analysis showed that the cavitation jet effectively removed crystal deposits from the surface of the articular cartilage and synovial membrane (Fig. 7A-C). The clearance ratio for crystal deposition within joints, on the cartilage surface, and the synovial surface, were significantly different when compared between the two groups (P = 0.0379/0.0109/0.0377, respectively). The cleaning rates exceeded 97% in all cavitation groups, which were 47.99 ± 9.63% higher than that in the control group; the 95% confidence interval was between 6.61% and 89.36%. The cleaning efficiency for articular cartilage in the experimental group was 27.50 ± 3.11% higher than that in the control group; the 95% confidence interval was between 14.65 and 40.35%. The cleaning rate for the synovial surface in the experimental group was 58.69 ± 11.80% higher than that in the control group; the 95% confidence interval was 8.181–109.2(Fig. 7C).

**Fig. 8:**
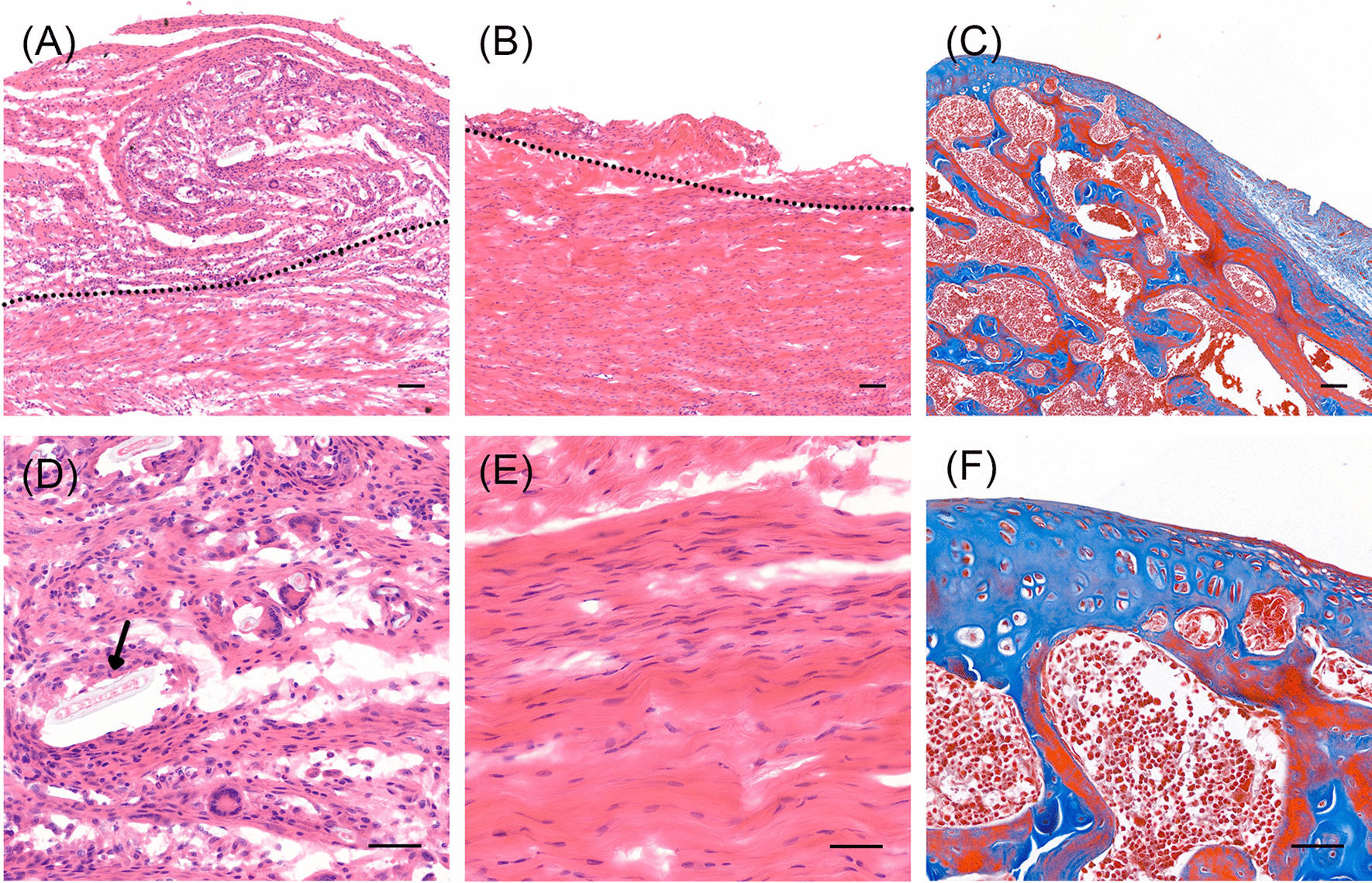
Histological evaluation of synovium tissues after the clearence experiment. Representative images showed the target region of cavitation bubbles. **A** Preoperative MSU-induced inflammatory region showed characterized disorderly collagen fibers and recruitment of inflammatory cells, distributed within 200 μm on one side exposed to synovial fluid. The dotted line repres ents the boundary between the inflammatory foci at the top and normal tissue at the bottom. **B** After debridement by cavitation devices, aligned collagen fibers in zone of normal tissue were preserved while most of the inflammatory areas were removed. **C** No impairment was observed in the cartilage and subchondral bone of the same joint with Masson staining. **D**–**F** Zoom- in image of (**A**–**C**), respectively. The black arrow shows deposition of MSU crystals in tissue. Scale bar: 100 μm **(A**–**C**), 50 μm (**D**–**F**)

We then analyzed the histological features of tissues in target area of cavitation jet equipment (Fig. 8). In control group, area with MSU deposition present as disordered collagenous fibers and aggregated inflammatory cells within 200 microns of the exposed synovial fluid (Fig. 8A). Enlarge image showing sodium urate crystals (black arrows) surrounded by collagen fibers and inflammatory cells (Fig. 8C). However, with debridement by cavitation jet equipment, the MSU deposition site full of infiltrating cells was absent (Fig. 8B). Zoom-in image showed that the deposition site was effectively removed, while normal collagen fibers are well preserved in orderly arrangement (Fig. 8D). Therefore, we verified that cavitation jet equipment could effectively remove deposition site under selected working condition without effecting adjacent normal tissues.

## Discussion

We have introduced a novel hydrodynamic cavitation device for the debridement of intra-articular MSU crystals. The feasibility and safety of this device was firstly verified in an animal model. Quantitative analysis further demonstrated that the cavitation jet system exceeded the cleaning efficiency of existing surgical instruments and effectively removed deposition from target sites while preserving surrounding normal collagen fibers.

Arthroscopic surgery represents an ideal approach for the diagnosis and treatment of crystal-deposited disease [16]. The device allows surgeons to directly observe and sample intra-articular structuresand is effective for the differential diagnosis of joint injury, arthritis, or tumors, especially for those with similar symptoms. Arthroscopy also provides us with options for the surgical treatment of tophaceous gouty arthritis and has several advantages over existing devices. A retrospective clinical study, including 173 patients, showed that the combination of febuxostat with arthroscopic surgery resulted in better outcomes than medication alone for patients with chronic gout [7]. However, existing surgical instruments for tissue dissection is time-consuming and can not fully remove MSU crystals from the surface of joints. The classic pathophysiological features of gouty arthritis is recognized as the deposition of crystals surrounded by immune cells and tissue cells on the surfaces of the synovium, tendons or cartilage [17, 18, 19]. When viewed by arthroscopy, such deposition is erosive and has no clear boundaries [20]. Therefore, a new surgical device is desired for the rapid removal of surface crystals that does not cause damage to the underlying tissues.

The cavitation effect refers to when the local absolute pressure of a certain place in the high-speed flow of water drops to the saturated vapor pressure at the local temperature; at this point, the air dissolved in the water will be released, forming cavitation bubbles, thus producing compression waves or micro-jets that exert significant effects on the nearby solid surface. This phenomenon has been widely applied in surgical procedures, such as ultrasonic ablation or urological surgeries; however, prior to this study, the application of this technique in arthroscopy had not been investigated [21, 22].

To generate cavitation more effectively, researchers have developed various forms of cavitation water jet nozzles, such as self-resonating cavitation nozzles and cross flow nozzles. Researchers have also investigated the influence of different types of nozzles and different working conditions on jet performance under submerged conditions [23]. Jesnitzer et al. reported that a nozzle with a conical angle of 60° and a cylindrical section of 0.5D had the best erosion effect under submerged conditions [24]. Subsequently, Vijay et al. investigated coaxial nozzles and found that when the jet was ejected from a nozzle at high speed, a shear action was produced with the surrounding stationary liquid or low-speed circulation, thus forming a vortex-cavitation water jet [25].

In this report, a special nozzle was designed and crystals removal efficiency of the equipment was verified while normal synovial and cartilage tissue well preserved. All joints were pretreated with saline to avoid bias from unattached crystals. For the tightly attached crystals or formed tophi, traditional surgical tools could remove a certain amount of MSU crystals, thus relieving the symptoms of patients [7]. This study have set up a comparison and found the cavitation jet device effectively exceeded the cleaning efficiency of the control group and remove stubbornly attached MSU that was surrounded by collagen fibers in tophi. By adjusting the appropriate parameters, the cavitation jet device could target the inflammatory sites caused by MSU crystals in connective tissues that is usually distributed within 200 µm on the side exposed to synovial fluid. Of note, rapid removal of crystals is of great clinical significance, which helps to reduce the operation time, thus reducing the occurrence of complications and speeding up the rehabilitation process [26]. Meanwhile, the jet pressure was set to be much lower than the maximum pressure on the normal articular surface(5Mpa) of the human knee joint,in order to avoid the risk of concurrent cartilage injuries [27].

There are some limitations in the present study that need to be considered. Firstly, the cavitation device has only been tested in rat models; further research should be carried out on larger animals or cadaver specimens to validate our results. Secondly, the cavitation device is currently used on a bench; the next step should aim to standardize equipments including handle and nozzle so that they can be matched to standard of instruments for endoscopes of arthroscopes and generate cavitation effects in the joint cavity. In particular, an elongated catheter between the nozzle and the handle is required to allow nozzle to pass through the surgical approach through the cannula so that it can be tested in vivo under anesthesia to further verify its potential for surgeries.

In conclusion, we have developed a hydrodynamic cavitation device for surgical use on patients with chronic gouty arthritis. We also demonstrated the feasibility and safety profile of this device in a rat model of gout. Our findings may open up new opportunities for the intraoperative treatment of chronic gouty arthritis and other MSU-related diseases.

## Data Availability

All data is available in the main text or the supplementary materials.
